# Spatial distribution modelling of *Culicoides* (Diptera: Ceratopogonidae) biting midges, potential vectors of African horse sickness and bluetongue viruses in Senegal

**DOI:** 10.1186/s13071-018-2920-7

**Published:** 2018-06-08

**Authors:** Maryam Diarra, Moussa Fall, Assane Gueye Fall, Aliou Diop, Renaud Lancelot, Momar Talla Seck, Ignace Rakotoarivony, Xavier Allène, Jérémy Bouyer, Hélène Guis

**Affiliations:** 1 InstitutSénégalais de Recherches Agricoles, Laboratoire National de l’Elevage et de Recherches Vétérinaires, Dakar, Sénégal; 2 Université Gaston Berger, Laboratoire d’Etudes et de Recherches en Statistiques et Développement, Saint-Louis, Sénégal; 3 Institut Pasteur de Dakar, G4 Biostatistique, Bioinformatique et Modélisation, Dakar, Sénégal; 4 CIRAD, ASTRE, Montpellier, France; 5 ASTRE, INRA, CIRAD, Univ Montpellier, Montpellier, France; 6 Cirad, ASTRE, Antananarivo, Madagascar; 7 Institut Pasteur, Epidemiology Unit, Antananarivo, Madagascar; 8 FOFIFA, DRZVP, Antananarivo, Madagascar

**Keywords:** Senegal, African horse sickness, *Culicoides* vectors, Environmental and climatic data, Random forest models, Generalized Linear Models, Spatial distribution, Mapping

## Abstract

**Background:**

In Senegal, the last epidemic of African horse sickness (AHS) occurred in 2007. The western part of the country (the Niayes area) concentrates modern farms with exotic horses of high value and was highly affected during the 2007 outbreak that has started in the area. Several studies were initiated in the Niayes area in order to better characterize *Culicoides* diversity, ecology and the impact of environmental and climatic data on dynamics of proven and suspected vectors. The aims of this study are to better understand the spatial distribution and diversity of *Culicoides* in Senegal and to map their abundance throughout the country.

**Methods:**

*Culicoides* data were obtained through a nationwide trapping campaign organized in 2012. Two successive collection nights were carried out in 96 sites in 12 (of 14) regions of Senegal at the end of the rainy season (between September and October) using OVI (Onderstepoort Veterinary Institute) light traps. Three different modeling approaches were compared: the first consists in a spatial interpolation by ordinary kriging of *Culicoides* abundance data. The two others consist in analyzing the relation between *Culicoides* abundance and environmental and climatic data to model abundance and investigate the environmental suitability; and were carried out by implementing generalized linear models and random forest models.

**Results:**

A total of 1,373,929 specimens of the genus *Culicoides* belonging to at least 32 different species were collected in 96 sites during the survey. According to the RF (random forest) models which provided better estimates of abundances than Generalized Linear Models (GLM) models, environmental and climatic variables that influence species abundance were identified. *Culicoides imicola*, *C. enderleini* and *C. miombo* were mostly driven by average rainfall and minimum and maximum normalized difference vegetation index. Abundance of *C. oxystoma* was mostly determined by average rainfall and day temperature. *Culicoides bolitinos* had a particular trend; the environmental and climatic variables above had a lesser impact on its abundance. RF model prediction maps for the first four species showed high abundance in southern Senegal and in the groundnut basin area, whereas *C. bolitinos* was present in southern Senegal, but in much lower abundance.

**Conclusions:**

Environmental and climatic variables of importance that influence the spatial distribution of species abundance were identified. It is now crucial to evaluate the vector competence of major species and then combine the vector densities with densities of horses to quantify the risk of transmission of AHS virus across the country.

**Electronic supplementary material:**

The online version of this article (10.1186/s13071-018-2920-7) contains supplementary material, which is available to authorized users.

## Background

African horse sickness (AHS) and Bluetongue (BT) are vector-borne viral diseases affecting equids and ruminants (mainly cattle and small ruminants), respectively. These two viruses are biologically transmitted by females of several species of *Culicoides* (Diptera: Ceratopogonidae) biting midges. Bluetongue is distributed worldwide, affecting almost all continents (except Antarctica) while AHS occurs in sub-Saharan Africa, with rare incursions into Europe and Asia [1–3]. The importance of these two arboviral diseases derives from their potential for rapid spread and their major economic impact due to direct mortalities, restriction of animal movements, surveillance and vaccination costs, as shown by the recent outbreaks of BT in Europe [4–6] and AHS in Africa [7, 8]. Their importance requires immediate notification to the World Animal Health Organization [9].

Following the important AHS outbreak in Senegal in 2007 [7, 8], several studies were initiated in the Niayes area (in the western part of the country) in order to better characterize *Culicoides* diversity and ecology [10–14], and the impact of environmental and climatic variables on the dynamics of major vector species (proven and suspected vectors) [15]. These studies recorded for the first time the presence of *Culicoides oxystoma* and showed that this species was extremely abundant in the Niayes, both in black-light and in horse-baited traps. Because of its abundance, close contact with horses and its known or suspected role in the transmission of other arboviruses (Akabane, epizootic haemorrhagic disease and bluetongue viruses), these studies concluded that it should be considered as a potential vector of AHS virus. These studies also showed that *Culicoides* population peak of abundance occurred at end of the rainy season in September and October.

In order to anticipate future events, prevent and better control *Culicoides*-borne disease outbreaks, it is essential to identify areas with higher risk of *Culicoides*-borne pathogens transmission at a national or regional level. This allows characterizing vector species distribution in Senegal. This is essential to determine if vectors are present throughout the country, if their abundance is highly variable or not and thus identify high-risk transmission areas. Combined with horse population distribution, this enables to target vaccination in these high-risk areas in the event of an outbreak. The first step towards this aim implies improving our knowledge on the distribution of vectors in Senegal. Thus, a nation-wide entomological trapping campaign was carried out to model the abundance of proven and potential vectors of AHS virus (AHSV) and/or BT virus (BTV) in Senegal and map their distribution.

Several studies demonstrated the ability to predict the presence and/or abundance of *Culicoides* midges using meteorological and environmental variables mainly derived from satellite imagery. These models were established using statistical techniques such as discriminant analysis [16–20], generalized linear models such as logistic regression [21–25] and more recently, data-mining techniques such as random forests [17, 26, 27].

The aim of this study is to model the spatial distribution of *Culicoides* species which are known or suspected to be vectors of AHSV and/or which are the most abundant in Senegal. The models could help stakeholders to make decisions for the control of these diseases. For this, entomological data were obtained through a nationwide trapping campaign and models for each species were developed to map their abundance. Three different modeling approaches were compared in order to find a simple yet robust method for each species. The first method consists in a spatial interpolation by ordinary kriging of *Culicoides* abundance data and does not necessitate environmental data. The other two consist of analyzing the relationship between *Culicoides* abundance, environmental and climatic data to model abundance in unsampled areas and were carried out by implementing generalized linear models and random forest models.

## Methods

### Entomological data

**Fig. 1:**
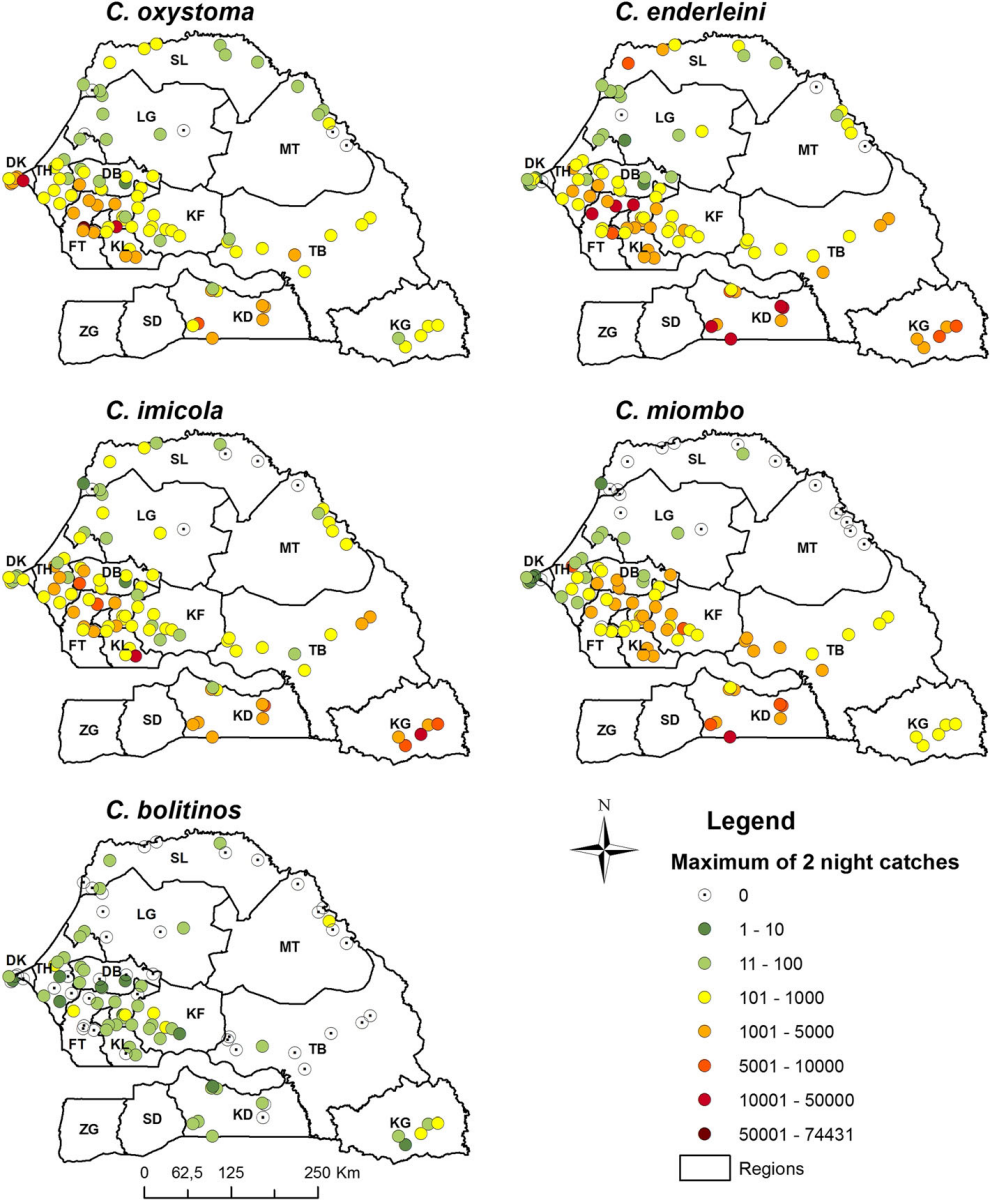
Maximum abundance maps (2 nights of captures) for the five *Culicoides* species of veterinary interest in Senegal. *Abbreviations*: DK, Dakar; TH, Thies; DB, Diourbel; FT, Fatick; KF, Kaffrine; KL, Kaolack; LG, Louga; SL, Saint-Louis; MT, Matam; TB, Tambacounda; KD, Kedougou; KL, Kolda; SD, Sedhiou; ZG, Ziguinchor

In 2012, a nationwide *Culicoides* trapping campaign was organized to collect information on the spatial distribution of *Culicoides* species in Senegal. Overall, with the help of the veterinary services, 108 sites holding equids were initially selected as follows: 3 sites per department and 3 departments per region in 12 (of the 14) regions of Senegal. Both the Institut Sénégalais de Recherches Agricoles (ISRA) team and veterinary services’ officers carried out trapping over two consecutive collection nights in each site at the end of the rainy season (between September and October). This timeframe was chosen because it corresponds to the peak of abundance of *Culicoides* in the Niayes region [13–15, 28]. Geographical coordinates of sampling sites (Fig. 1) were recorded using Garmin© global positioning system receivers (accurate to within 10 meters). *Culicoides* species were collected using Onderstepoort black-light suction traps (Onderstepoort Veterinary Institute South Africa) positioned close to equids (for further information on trap set up see [13, 14]). Identification of *Culicoides* species was carried out as explained by [13].

Maximum abundance of the two nights was considered as the best estimate of the population present as abundance can decrease rapidly in sub-optimal trapping conditions [29]. If the mean abundance between two nights is used instead of the maximum abundance, then the abundance recorded will be decreased in the sites. Mean is typically meaningful when data are normally distributed. Maximum is much more meaningful here as we are sure that the site holds at least this number of *Culicoides* corrected by an unknown trap efficiency factor. Entomological data are provided in Additional file 1: Table S1.

### Climatic and environmental data

Climatic and environmental variables characterizing favorable habitats for *Culicoides* were selected based on literature review of presence and abundance models [15–18, 20, 26, 30–37]. A total of 21 variables were selected belonging to 5 categories (temperature, vegetation index, precipitation, land cover and livestock density). Climatic variables included day-time and night-time land surface temperature (DLST and NLST) and rainfall. Environmental variables included the normalized difference vegetation index (NDVI), percentages of 3 land-cover classes (water, savannah and forest), and ruminant host densities.

Temperature, NDVI and rainfall were extracted from satellite images from September 1, 2011 to October 31, 2012. The DLST and NLST images were obtained from MODIS sensor (http://modis.gsfc.nasa.gov) and NDVI images were derived from Spot-Vegetation (https://rs.vito.be/africa/en/data/Pages/vegetation.aspx). For DLST, NLST and NDVI, the maxima, minima and means were calculated for this 14 months period. Precipitation data were derived from National Oceanic and Atmospheric Administration (http://iridl.ldeo.columbia.edu/expert/SOURCES/.NOAA/.NCEP/.CPC/.FEWS/.Africa/.DAILY/.ARC2/.daily/.est_prcp/). The maximum, mean and cumulative rainfalls were calculated. Land cover data was derived from the Senegal Land Cover Database produced by the Food and Agriculture Organization of the United Nations (FAO) in the framework of Global Land Cover Network activities (http://www.fao.org/geonetwork/srv/en/main.home?uuid=545be438-bc87-480b-83ec-ba3f4e486daf). Percentage of surface covered by water, particularly favorable for *Culicoides* [15, 26] savannah and forest in the 1 km^2^ pixels comprising the trap were extracted. Ruminant (cattle, sheep and goats) livestock density data was obtained from the global distribution of livestock maps produced by the FAO (http://www.fao.org/ag/againfo/resources/en/glw/GLW_dens.html).

All data layers were clipped on the Senegalese territory and projected in the same projection system with a spatial resolution of 1 km^2^ using R2.10.1 statistical language environment [38] using of the R-package: *maps* [39], *mapdata* [40], *maptools* [41], *raster* [42], *rgdal* [43] and *sp* [44]. Climatic data are provided in Additional file 2: Figure S1.

### Statistical analysis

Three methods were used to map the abundances of targeted species of interest: ordinary kriging and two regression techniques: generalized linear models and random forests.

### Spatial interpolation by ordinary kriging

Ordinary kriging is an interpolation technique that aims to predict the value of a variable in an unsampled site [45] taking into account the spatial dependence of the data. It enables converting point data (data measured in specific sample sites) into raster data (images). In spatial statistics, the empirical semivariance is described as follows:

$$ \gamma\ (h)=\frac{1}{2}.\frac{1}{N(h)}\sum \limits_{i=1}^{N(h)}\Big({\left(z\left({x}_i+h\right)-z\left({x}_i\right)\right)}^2 $$

where, *z* (*x*_*i*_) are data at a particular location, *h* is a distance between ordered data, *N*(*h*) the number of paired data at a distance of *h*.

First experimental semi-variograms (a graph of semivariances versus distances between ordered data) are plotted to visualize statistical dependence values. Then a model is adjusted according to semivariogram's points to assess the statistical dependencies between sites and thus determine the maximal interpolation distance (over this distance interpolations can no longer be carried out).

### Generalized linear model

One of the distributions commonly used to model count data is the Poisson distribution. However, this distribution assumes equidispersion of count data. *Culicoides* data generally show strong overdispersion [15]. Overdispersion was tested according to [46]. The statistical approach was planned as follows: in case of significant overdispersion of the residuals of the Poisson model, a negative binomial model was tested, and in case again of significant overdispersion of residuals of the negative binomial model, a hurdle model was tested [47, 48].

### Random Forest model

Random Forest (RF) method is a robust ensemble learning technique for data analysis which consists of a set of classifications and regressions trees constructed from sub-samples of the complete data set [49]. This approach can be applied to model either the presence probability by performing classifications for qualitative variables (Random Forest classification) [17, 26, 27] or abundances by performing regressions for quantitative variables (Random Regression Forest) [26]. For more information on RF models and their application to model *Culicoides* presence and abundance, see [26]. By bootstrapping the data and by randomly changing the predictive variable sets over the different tree, RF increase diversity among regression trees. Each of the k regression trees is grown using another bootstrap subset of the original data set and the nodes are split using the best split variable among a subset of m randomly selected predictive variables [50]. RF parameters, which are the number of trees (k) and the number of predictive variables used to split the nodes (m), are user-defined. In this study, to allow for error convergence, k was set to 500 and m to 4.

The Root means squared error (RMSE) was calculated for internal validation of general linear models (GLM) and RF models. Statistical analysis and modeling were performed with the R2.10.1 statistical language environment [38] using of the R-package: *rgdal* [43], *splancs* [51], *gstat* [52], *maptools* [41] and *randomForest* [50].

## Results

Between September, 17th and October, 14th 2012, among the 108 sites initially selected, 98 sites were sampled and 10 sites were not prospected because of logistical issues. In two of the 98 sampled sites the trapping results were not exploitable and thus were not considered (one conservation issue and one battery failure).

A total of 1,373,929 specimens of the genus *Culicoides* belonging to 32 different species were collected in 96 sites during the survey (Table 1). At least 32 species were collected; there may be other species among the 1.8% of captured *Culicoides* that were not identified. Among those specimens, some belong to the Milnei and Similis groups but could not be identified more precisely as one sex was missing. Some individuals belonging to the Imicola group were shown to world experts of *Culicoides* but could not be identified; at this stage it is unclear whether they are variants of known species or new species. Two species were recorded for the first time in Senegal: *Culicoides murtalai* [53] and *Culicoides ochrothorax* [54], increasing the number of described *Culicoides* species found in Senegal from 53 [14] to 55.

**Table 1:** Abundance and frequency of *Culicoides* species captured during the nationwide survey in Senegal

Species	Total no. collected^a^	Mean abundance per site/per night	% of catches	% of females	No. of positive sites (%)
*C. oxystoma* ^b^	369,618	1925.04	26.9	85.46	93 (96.88)
*C. enderleini* ^b^	328,339	1710.09	23.89	86.79	92 (95.83)
*C. imicola* ^b^	197,573	1029.02	14.38	86.95	92 (95.83)
*C. miombo* ^b^	176,917	921.44	12.87	87.80	79 (82.29)
*C. trifasciellus*	147,917	770.40	10.76	97.76	54 (56.25)
*C. milnei*	30,784	160.33	2.24	94.55	38 (39.58)
*C. neavei*	29,958	156.03	2.18	96.91	27 (28.13)
*C. kingi*	16,706	87.01	1.21	79.82	41 (42.71)
*C. moreli*	14,978	78.01	1.09	85.69	54 (56.25)
*C. leucostictus*	7277	37.90	0.53	74.71	53 (55.21)
*C. nevilli*	6345	33.04	0.46	49.16	33 (34.38)
*C. distinctipennis*	5131	26.72	0.37	70.10	25 (26.04)
*C. quinquelineatus*	4783	24.91	0.34	91.26	21 (21.88)
*C. bolitinos* ^b^	4460	23.23	0.13	83.97	55 (57.29)
*C. nivosus*	1894	9.86	0.09	83.54	34 (35.42)
*C. fulvithorax*	1366	7.11	0.08	97.91	17 (17.71)
*C. pseudopallidipennis*	1198	6.24	0.04	93.29	13 (13.54)
*C. translucens*	614	3.19	0.03	34.56	14 (14.58)
*C. accraensis*	504	2.62	0.03	58.86	19 (19.79)
*C. hortensis*	481	2.50	0.03	96.40	6 (6.25)
*C. murtalai*	481	2.50	0.01	100	2 (2.08)
*C. similis*	260	1.35	0.01	65.21	11 (11.46)
*C. pycnostictus*	228	1.18	< 0.01	94.60	4 (4.17)
*C. africanus*	91	0.47	< 0.01	100	4 (4.17)
*C. austeni*	78	0.40	< 0.01	100	2 (2.08)
*C. azerbajdzhanicus/C. ravus*	57	0.29	< 0.01	98.25	2 (2.08)
*C. punctithorax*	45	0.23	< 0.01	4.48	2 (2.08)
*C. ochrothorax*	42	0.21	< 0.01	100	1 (1.04)
*C. yankari*	32	0.16	< 0.01	100	1 (1.04)
*C. exspectator*	26	0.13	< 0.01	100	1 (1.04)
*C. vomensis*	18	0.09	< 0.01	100	2 (2.08)
*C. robini*	1	0.005	< 0.01	100	1 (1.04)
*Culicoides* sp.	25,729	134.00	1.81	73.27	–
Total	1,373,929	7155.88	100	87.90	–

^a^2 nights, 96 sites

^b^The five species of veterinary interest selected

Each of the following five species represented more than 10% of the catch: *Culicoides oxystoma*, *Culicoides enderleini*, *Culicoides imicola*, *Culicoides miombo* and *Culicoides trifasciellus*. As found previously in the Niayes region, *C. oxystoma* (26.9% of catches) was the most abundant species and was present in 93 out of the 96 sites sampled (97% of sites). Percentages of individuals caught compared to the total (and frequencies of sites where the species was present) catches were 23.89% (95.83%) for *C. enderleini*; 14.38% (95.83%) for *C. imicola*; 12.87% (82.29%) for *C. miombo* and 10.76% (56.25%) for *C. trifasciellus*, respectively (Table 1).

We chose to model the distribution of five species: the first four most abundant (*C. oxystoma*, *C. enderleini*, *C. imicola* and *C. miombo*) and also *Culicoides bolitinos* as it is a proven vector of AHSV in South Africa [55]. *Culicoides bolitinos* was collected in low numbers (0.32% of catches) and in 57.29% of the prospected sites.

For the five *Culicoides* species of veterinary interest, observed abundance data (maximum abundance of the two consecutive nights) are shown in Fig. 1. Overall, three species had similar (yet not identical) patterns: *C. oxystoma*, *C. imicola* and *C. enderleini*. They were abundant in the west (groundnut basin), in the south-east and in some sites in the north-east (river delta). The distribution of *C. miombo* differed mainly in that it was very rare in the north-eastern sites while the distribution of *C. bolitinos* was much sparser.

**Fig. 2:**
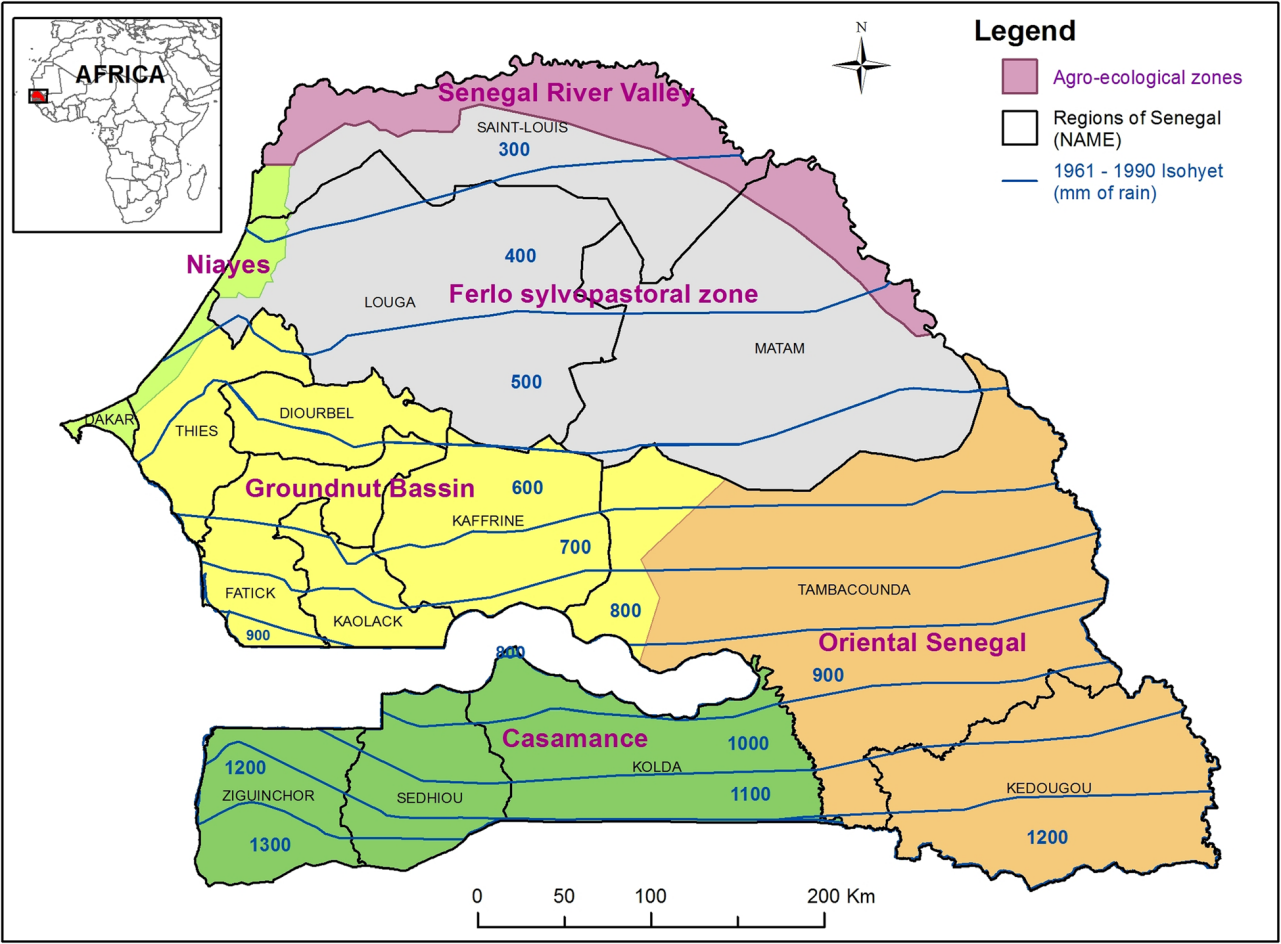
Representation of isohyets (1961–1990) and delimitation of agroecological zones of Senegal. Source: Adapted from Centre de Suivi Ecologique du Sénégal (CSE)

Regarding *C. oxystoma*, the sites where abundances were the highest are located in west central (Fatick, Kaolack and Diourbel regions), in the far west (Dakar region) and south of Senegal (Kolda region) (Fig. 2). For *C. enderleini*, the highest abundances were observed in the south (Kolda and Kedougou), in the groundnut basin (specifically in Fatick and Kaolack) and northwest of the Saint-Louis. For *C. imicola*, the highest abundances were recorded in the south and southeast (Kolda, Tambacounda and Kedougou) and in the center-west (Thies, Diourbel, Fatick and Kaolack). *Culicoides miombo* and *C. bolitinos* were very rare in northern Senegal. The highest abundances of *C. miombo* were obtained in Kolda, Tambacounda and in the groundnut basin (Fig. 2). *Culicoides bolitinos* abundances were very low. *Culicoides bolitinos* highest densities were noticed in Kedougou region and in the groundnut basin (particularly in Fatick, Kaffrine and Kaolack).

**Fig. 3:**
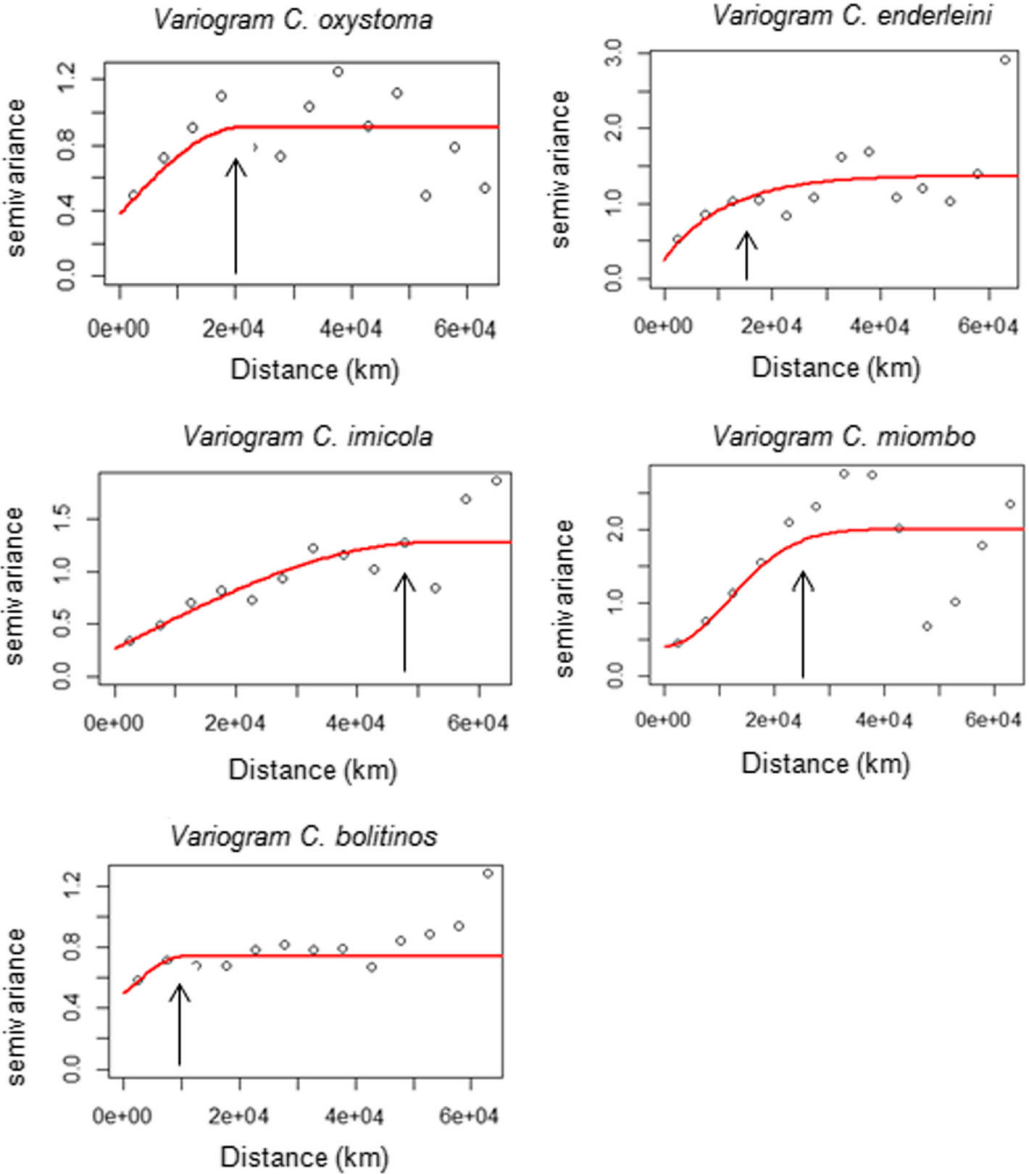
Variogram models for each of the five *Culicoides* species of veterinary interest in Senegal

**Fig. 4:**
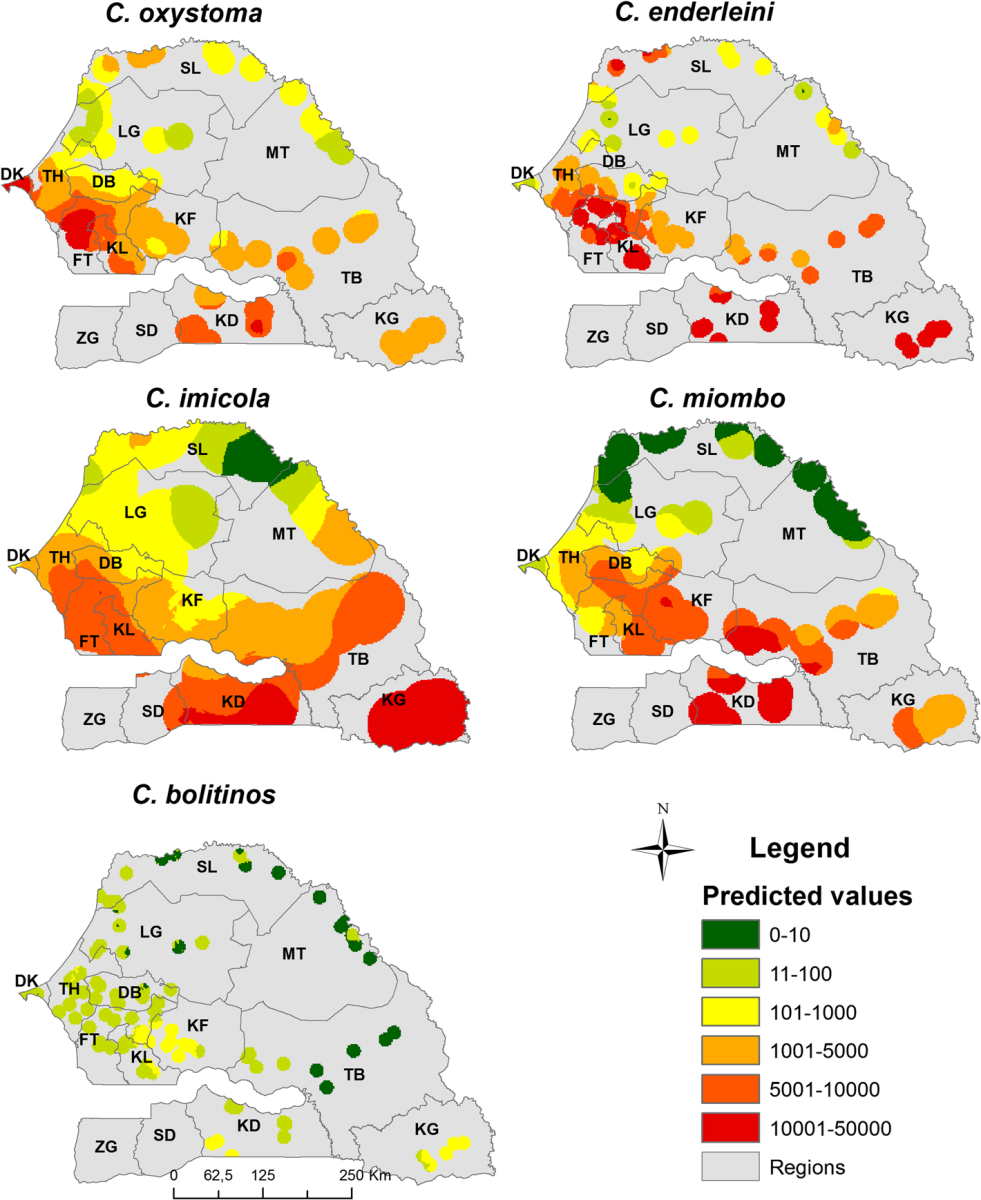
Abundance maps obtained by ordinary kriging for the five *Culicoides* species in Senegal. *Abbreviations*: DK, Dakar; TH, Thies; DB, Diourbel; FT, Fatick; KF, Kaffrine; KL, Kaolack; LG, Louga; SL, Saint-Louis; MT, Matam; TB, Tambacounda; KD, Kedougou; KL, Kolda; SD, Sedhiou; ZG, Ziguinchor

The variogram results determined the maximal interpolation distances for each of the five species. These distances were: 20 km for *C. oxystoma*, 48 km for *C. imicola*, 13 km for *C. enderleini*, 25 km for *C. miombo* and 10 km for *C. bolitinos* (Fig. 3). Different models were fitted to the variograms: spherical models for *C. oxystoma*, *C. imicola* and *C. bolitinos*, a circular model for *C. miombo* and an exponential model for *C. enderleini*. Abundance maps by ordinary kriging were developed respecting the maximal interpolation distance for each species (Fig. 4). For *C. oxystoma*, *C. enderleini*, *C. imicola* and *C. miombo*, abundances were predicted to be very high in west-central and southern Senegal and decrease gradually towards the north. However, high abundances of *C. enderleini* were predicted in the northwest of Senegal and, to a lesser extent, for *C. oxystoma*. For *C. bolitinos*, the highest abundances were modelled in the extreme south of Senegal and south of the groundnut basin (specifically in Kaffrine region).

**Fig. 5:**
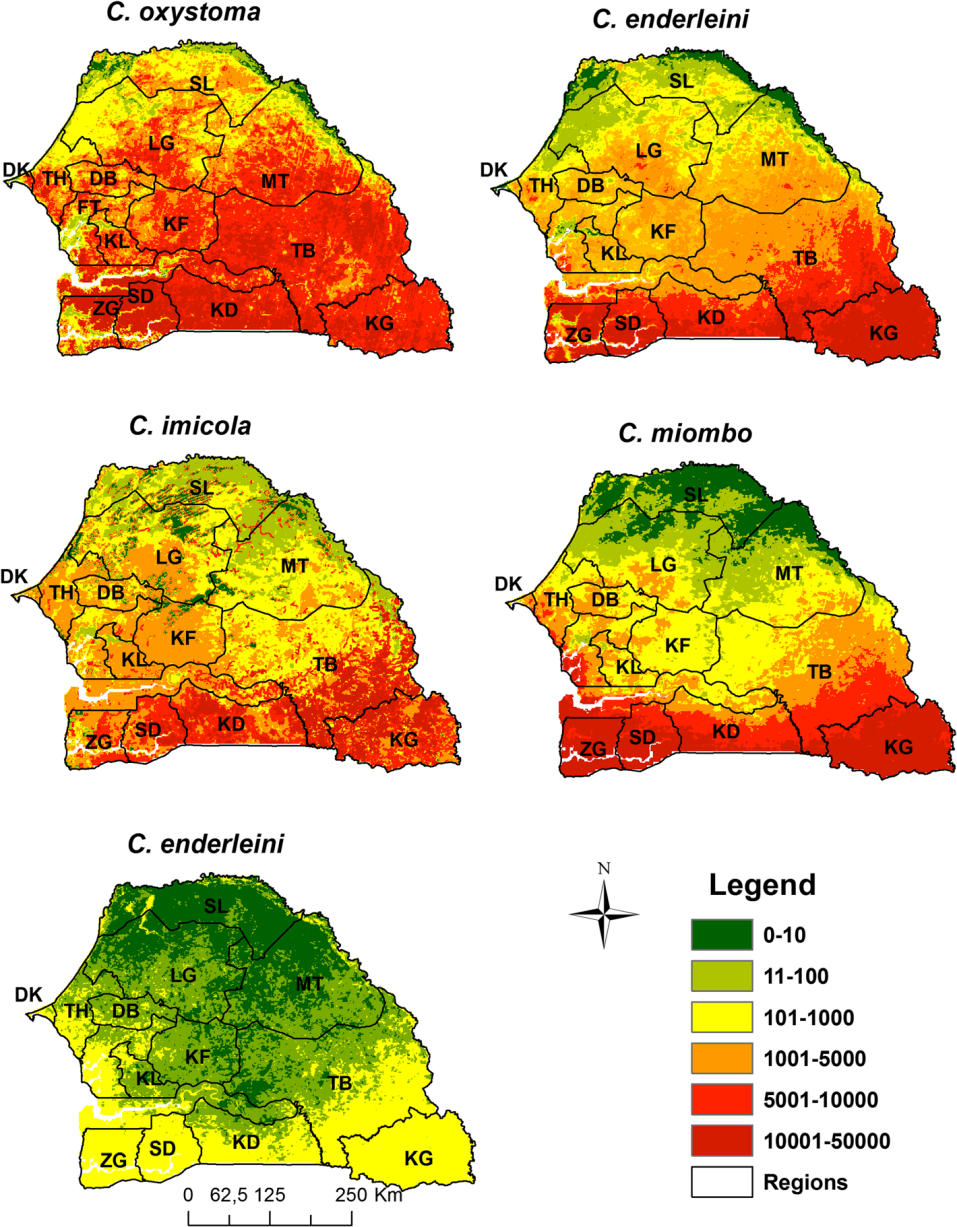
Predicted abundance maps according GLM model for five *Culicoides* species of veterinary interest in Senegal. *Abbreviations*: DK, Dakar; TH, Thies; DB, Diourbel; FT, Fatick; KF, Kaffrine; KL, Kaolack; LG, Louga; SL, Saint-Louis; MT, Matam; TB, Tambacounda; KD, Kedougou; KL, Kolda; SD, Sedhiou; ZG, Ziguinchor

To produce abundance maps for the entire Senegalese territory, GLM and RF models were developed. Table 2 presents the 10 most important predictors for each species according to GLM models. Maximum NDVI was among the three most important variables for all species. Average rainfall was also among the 3 most important variables for 3 of the 5 species (*C.* o*xystoma*, *C. enderleini* and *C. imicola*). Other environmental variables that influence *Culicoides* abundances were day and night temperatures and percentage cover of water bodies for *C. oxystoma* and *C. imicola*; night temperatures for *C. enderleini*; day and night temperatures for *C. miombo*; livestock density and percentage cover of savannah for *C. bolitinos*. Abundance maps based on GLM modeling showed that for all five species of veterinary interest, predicted abundances are very low along the Senegal River Valley and very high in the south (Fig. 5). High abundances of *C. imicola* and *C. oxystoma* are predicted on the entire country although for *C. imicola* areas of high and low abundance are strongly interlinked (areas of high abundance are often close to areas of low abundance). *Culicoides imicola* and *C. enderleini* predicted abundances are particularly strong in the southern third of the country and medium in the middle third. The distribution of *C. miombo* in the south is even more pronounced: abundant in the southern third, it is less abundant in the central and almost absent in the northern third of the country. Not surprisingly, *C. bolitinos* predicted abundances are lower than those of the other species. The area where it is most abundant is the delta in Casamance in southeast Senegal and in southwest Senegal (Fig. 5).

**Table 2:** The 10 most important predictors according to GLM model, indicated by the variable importance (VarImp) for the five *Culicoides* species of veterinary interest

*C. oxystoma*		*C. enderleini*		*C. imicola*		*C. miombo*		*C. bolitinos*	
Predictors	VarImp	Predictor	VarImp	Predictor	VarImp	Predictor	VarImp	Predictor	VarImp
AvRain^a^	83	MaxNDVI^a^	100	AvRain^a^	74	MaxNDVI^a^	100	MaxNDVI^a^	72
MaxNDVI^a^	74	AvRain^a^	64	MinNDVI^a^	58	MinNDVI^a^	98	AvHost^a^	64
MinDlst^a^	62	MinNDVI^a^	58	MaxNDVI	34	MaxNlst^a^	87	lcSavanha^a^	63
MaxNlst^a^	57	MaxNlst	39	MaxNlst	32	MinDlst^a^	68	MaxDlst	47
MinNlst	37	MinNlst	33	MinDlst	32	AvHost	37	MaxNlst	36
lcWater	35	MaxRain	28	lcWater	28	AvRain	36	avDlst	30
MinNDVI	22	lcWater	17	avNlst	27	AvDlst	21	lcForest	16
MaxRain	18	AvDlst	13	avDlst	27	MaxRain	15	lcWater	14
AvDlst	14	lcForest	11	AvHost	25	AvRain	12	AvRain	10
AvHost	12	MinDlst	8	MaxRain	23	lcWater	8	MinDlst	6

*Abbreviations*: *AV* average, *Min* minimum, *Max* maximum, *Dlst* day land surface temperature, *Nlst* night land surface temperature, *lc* landcover, *NDVI* normalized difference vegetation index, *Host* livestock density

^a^ Predictors for which VarImp are greater than 50%

Table 3 lists the 10 most important predictor variables from RF model of the different *Culicoides* species. Rainfall and/or NDVI were the most important variables influencing the abundance of the five *Culicoides* species. This is particularly true for *C. imicola*, *C. enderleini* and *C. miombo*, since the 3 most important variables are average rainfall and minimum and maximum NDVI. Abundance of *C. oxystoma* was mostly determined by average rainfall and day temperature and less so by NDVI (only ranked 7th by order of importance). Abundance of *C. enderleini* was mostly determined by average precipitation, NDVI (minimum and maximum) and average day temperature; that of *C. imicola* by average precipitation and maximum NDVI. Abundance of *C. miombo* was mostly driven by maximum NDVI followed by average precipitation, minimum NDVI and average night temperature. *Culicoides bolitinos* had a particular trend; the above environmental and climatic variables had a lesser impact on its abundance.

**Table 3:** The 10 most important predictors according to the RF model, indicated by the variable importance (VarImp) for the five *Culicoides* species of veterinary interest

*C. oxystoma*		*C. enderleini*		*C. imicola*		*C. miombo*		*C. bolitinos*	
Predictor	VarImp	Predictor	VarImp	Predictor	VarImp	Predictor	VarImp	Predictor	VarImp
AvRain^b^	13.54	AvRain^b^	13.40	AvRain^b^	18.82	MaxNDVI^a^	47.42	MaxNDVI^c^	6.31
AvDlst^b^	11.41	MinNDVI^b^	13.16	MaxNDVI^b^	10.37	AvRain^b^	20.64	AvHost^c^	5.91
MaxDlst^c^	6.07	MaxNDVI^b^	11.87	MinNDVI	8.42	MinNDVI^b^	11.15	MinDlst^c^	5.66
MinNlst^c^	5.96	AvDlst^b^	11.08	MaxRain^c^	6.19	AvNlst^b^	10.51	MaxDlst^c^	5.65
AvNlst^c^	5.47	MaxDlst^c^	8.13	AvNlst^c^	5.66	MaxNlst^c^	9.50	AvDlst^c^	5.64
MinDlst^c^	5.32	MaxRain^c^	6.35	AvDlst^c^	5.25	MaxDlst^c^	8.88	MaxNlst^c^	5.30
MaxNDVI^c^	5.04	MinNlst^c^	5.85	MinNlst	4.62	MinNlst^c^	8.33	MinNDVI^c^	5.21
MaxRain	4.93	MinDlst^c^	5.31	MaxNlst	4.37	MaxRain^c^	7.25	lcSavanha^c^	5.20
MaxNlst	4.63	AvNlst^c^	5.16	MinDlst	4.09	MinDlst^c^	6.91	lcForest^c^	5.13
AvHost	4.08	AvHost	4.43	MaxDlst	3.45	AvDlst^c^	6.81	lcWater	4.72

^a^Predictors for which VarImp are greater than 30%

^b^Predictors for which VarImp are lower than 30% and greater than 10%

^c^Predictors for which VarImp are lower than 10% and greater than 5%

**Fig. 6:**
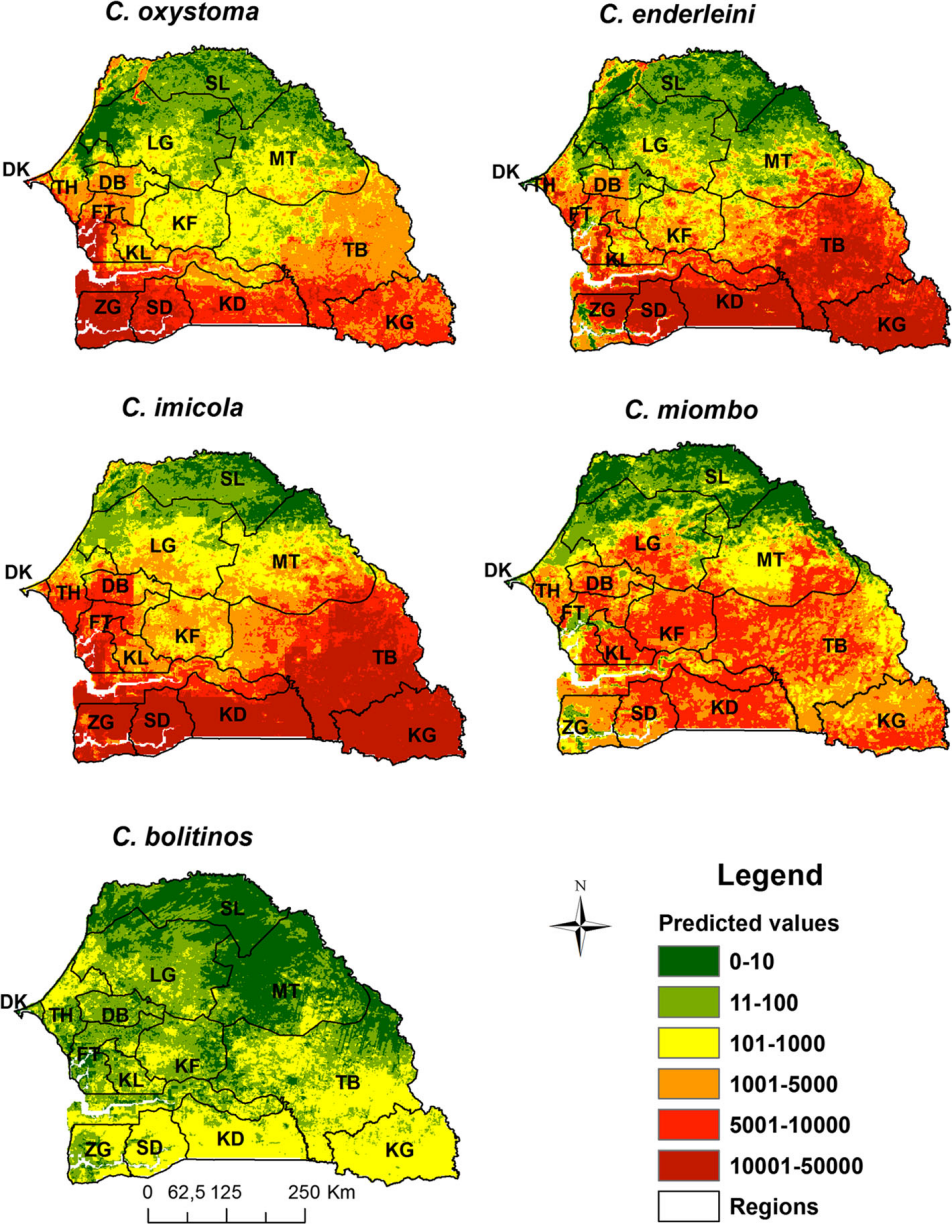
Predicted abundance maps according RF model for five *Culicoides* species of veterinary interest in Senegal. *Abbreviations*: DK, Dakar; TH, Thies; DB, Diourbel; FT, Fatick; KF, Kaffrine; KL, Kaolack; LG, Louga; SL, Saint-Louis; MT, Matam; TB, Tambacounda; KD, Kedougou; KL, Kolda; SD, Sedhiou; ZG, Ziguinchor

Predicted *Culicoides* abundance maps provided by RF model are shown in Fig. 6. For the five species, an increasing abundance gradient from north to south is predicted. For *C. oxystoma*, *C. enderleini* and *C. imicola*, predicted abundances are very high in the southern third of the country, and for *C. miombo* abundances are high to very high in the southern and middle thirds. For all the species, predicted abundances are very low in the north of the country, particularly in the north of Ferlo and in the Senegal River valley. *Culicoides oxystoma* is predicted to be particularly abundant along the western coast from Dakar to Ziguinchor and in The Gambia along the river. This species also has very high predicted abundances in the south, southeast and center-west of Senegal (Fig. 6). *Culicoides enderleini* and *C. imicola* exhibit a similar pattern with very high abundances predicted in Casamance (Ziguinchor, Sedhiou and Kolda), eastern Senegal (Tambacounda and Kedougou) and the center-west of the groundnut basin (Fatick and surrounding). Regarding *C. miombo* and *C. bolitinos*, very low abundances were predicted in the Niayes’ area. *Culicoides bolitinos* predicted abundances are lower than those of other species; the area where its abundance is predicted to be the highest is the south and southeast, particularly in Kedougou region.

Prediction errors measured by RMSE (on log_10_(n+1) scale) between observed and predicted values are lower for the RF model than for the GLM model for all five species (Table 4). Among RF models, predictions errors are lowest for *C. imicola* (RMSE = 0.31).

**Table 4:** Internal validation (root means squared error (RMSE) on log_10_(n+1) scale)of GLM and RF models for each of the five species of veterinary interest

Species	RMSE	
	GLM	RF
*C. oxystoma*	1.72	0.33
*C. enderleini*	1.9	0.37
*C. imicola*	1.75	0.31
*C. miombo*	1.36	0.37
*C. bolitinos*	1.42	0.38

## Discussion

The identification of suitable areas for five targeted *Culicoides* species in Senegal was performed with three different modelling techniques: a spatial interpolation method, kriging and two statistical modelling methods using meteorological and environmental variables, GLM and RF models. Interpolation by kriging yielded incomplete prediction maps. To obtain complete maps, more intensive entomological survey would be necessary, especially for *C. bolitinos* and *C. enderleini*. Given the logistical efforts required to conduct such survey and identify *Culicoides* species, this option seems unrealistic.

The comparison of predicted abundance maps from each model showed consistent spatial structures. Although the predictions of maximum abundances provided by kriging were limited by extrapolation distances, abundance maps by kriging and RF models identified similar structures whereas GLM models provided abundance maps slightly different from those resulting from kriging and RF models. GLM outcomes differed for *C. oxystoma* particularly in Tambacounda and Kedougou, for *C. enderleini* in Fatick, Kaffrine, Kaolack and western Saint-Louis, for *C. imicola* in Thies, Fatick, Kaolack, for *C. miombo* in Dakar, Kaffrine and Tambacounda and for *C. bolitinos* in Kaolack, Kaffrine and Fatick.

Prediction errors between observed and predicted abundances were lower for the RF model than for the GLM model. Recent studies have also shown that RF approaches provide good modelling results both for regression [26] and classification [17, 26, 27] trees. The effect of climatic and environmental variables will be discussed for each species based on the results from RF models since they provided better estimates of abundances.

Regarding *C. oxystoma*, this is, to our knowledge, the first spatial distribution model published. Studies on *Culicoides* dynamics in Senegal show that this species is very frequent and abundant in the Niayes area [13, 14]. Our study shows that this species is widespread and abundant over the entire country, with an increasing gradient from north to south, and high predicted abundance in the west, near the coast and around river deltas.

Average rainfall was the most important variable influencing *C. oxystoma*, *C. enderleini* and *C. imicola* abundance, and the second most important variable for *C. miombo*. This variable seems to limit the abundance of these species in the north, more arid (see isohyets representation of Senegal in Fig. 2). In addition to rainfall, day and night temperatures strongly influenced *C. oxystoma* abundance. Its highest abundance was observed in the groundnut basin and southern Senegal. Although ecological drivers of *C. oxystoma* remain poorly described, this species is known to be abundant in South Korea [56] and in Kagoshima, southern Japan [57]. These two areas are characterized by a humid subtropical climate. *Culicoides oxystoma* was collected from May to October in the south of Korea [56] and from May to November in southern Japan [57], i.e. in the warmer and rainy period of the year, showing that rainfall and temperature appear to influence the distribution of this species in both Japan and in Senegal.

Livestock density seems to have a weak influence on *C. oxystoma* abundances. Generally, livestock density was not identified as a variable with a high impact on *Culicoides* abundance except for *C. bolitinos*. This is surprising because for many other *Culicoides* species, especially those vectors of pathogens to animals, the presence of hosts and their density are key factors known to affect their catch [58–60]. Several reasons might explain this outcome. First, host densities data was available at a low spatial resolution (100 ×100 km). Secondly, these consisted in 2005 predictions, and densities could have changed (increased or decreased) since then. Thirdly, host densities included only three ruminant species (cattle, sheep and goats) but did not include horse densities. To our knowledge there is no map of horse densities available for Senegal. The lack of data on horse densities could be an important hindrance since in the field, in Senegal, Fall et al. [61] found high abundances of *C. oxystoma* on horses. Finally, some *Culicoides* species are able to feed on other hosts, including birds and wildlife, and it is possible that the densities of these other host species impact the distribution of that species.

As for *C. oxystoma*, little information in the literature is available on climatic and environmental variables that could impact *C. enderleini*. In this study, *C. enderleini* was very abundant in the southern third of the country. RF models showed that its abundance is significantly associated with rainfall, vegetation index, day and night temperatures and, to a lesser extent, livestock density. This is coherent since in the south of Senegal, rainfall is important, average NDVI, temperatures and livestock density are high.

The distribution of *C. imicola* and *C. enderleini* followed a similar pattern with an increasing north-south gradient. Again, average rainfall could be a limiting factor in the north, explaining higher abundances in the south (Ziguinchor, Kolda and Kedougou) and center-west (in the groundnut basin) regions that record the highest rainfall in Senegal. The importance of rainfall on the distribution and abundance of *C. imicola* was highlighted in Morocco [33] and Europe [22, 26, 37, 62]. The study of *Culicoides* dynamics in the Niayes area also showed that peaks of abundance of *C. imicola* occurred during the end of the rainy season (September-October) [13].

Maximum and minimum NDVI were the second and third most important variables associated with *C. imicola* abundance. The link between the distribution of *C. imicola* and NDVI is consistent with previous studies showing that high NDVI values were associated with increased *C. imicola* abundance in the Niayes area in Senegal [15], in north Africa and in Europe [16, 24, 30–33]. In Senegal, areas where the vegetation index is the highest are also those where rainfall is highest, as both are linked [63]. Studies have also highlighted the link between NDVI and soil moisture [64]. Soil moisture could be an important factor influencing larval development of this species. Temperatures (day and night) were also found associated with *C. imicola* abundance. This finding has been confirmed by several other studies [16, 18, 19, 25, 29, 32, 35].

Concerning *C. miombo*, maximum NDVI was by far the most important variable influencing its abundance. Its highest abundances were recorded in areas where the vegetation index is the highest, i.e. in the groundnut basin and in southern Senegal. In the north of the country where the vegetation index is the lowest, *C. miombo* is almost absent. In the Niayes area (particularly in Dakar and Thies), the abundance of *C. miombo* was low, confirming the results of a previous study [13]. This is probably due to the low vegetation index in this area. In this study, rainfall and temperatures were found associated with the abundance of *C. miombo*, in coherence with Meiswinkel’s study, which revealed that this species is sensitive to rainfall and high temperatures [65].

Regarding *C. bolitinos*, no single variable was a dominant driver of its abundance. Indeed, the contribution of the best environmental predictor (maximum NDVI) on its abundance remained very low (variable importance = 6.31). The most important variables impacting its abundance were the NDVI, livestock density and temperature. These three types of variables have also been linked to *C. bolitinos* abundance in South Africa [66]. Livestock density was an important variable which greatly impacted model accuracy. Studies conducted by Fall et al. [61] showed that *C. bolitinos* is particularly aggressive on horses with a blood-feeding rate of over 75%. This species is also known to feed on large mammals [67]. Other studies describe the larval habitats of *C. bolitinos* as being the dung of wild and domestic *Bovidae* in Africa [68]. The ecology of this species is therefore closely linked to its hosts (through feeding and through its breeding media) and it is thus coherent to find a strong link to livestock densities and weaker environmental drivers of its abundance.

Overall, the highest abundances of these five species, proven or suspected vectors of AHS and BT viruses, were recorded in southern Senegal. If *C. oxystoma* seems present rather in the southwest of the country, *C. enderleini*, *C. miombo* and *C. imicola* have very high abundances in the center-south (Kolda), in the south-east (Kedougou) and in the groundnut basin. *Culicoides* abundance maps from RF models were compared with those of AHS outbreaks in 2007 in Senegal [8], which show significant mortality of horses in the groundnut basin. According to Diouf et al. [8], the greatest risk of introduction of AHS virus in Senegal is through the northeastern border because of important commercial trade movements and numerous markets to and from which carters transport goods. These authors suggested focusing surveillance of AHS virus introduction in that area in order to prevent the virus from reaching the groundnut basin where the dense network of markets could substantially amplify disease transmission and diffusion. Diouf et al. [8] considered the south and southeastern part of the country to be less at risk of AHS because of lower horse densities due to the presence of tsetse flies. Because low densities of *Culicoides* were found in the north-eastern part of Senegal, this study strengthens the hypothesis that the main driver of AHS introduction and spread in 2007 was horse movements. It confirms that the groundnut basin is an area at high risk of AHS transmission, because it combines important vector, host and market densities, which could lead to important epidemics. The very important densities of *Culicoides* in the south suggest that if the virus was present in the neighboring countries (Mali, the Republic of Guinea and Guinea Bissau), it could be introduced through the spread of infected *Culicoides* and then be amplified in donkeys.

The two main knowledge gaps to better assess AHS transmission risk in Senegal are (i) vector competence of suspected vectors and of very abundant species; and (ii) a map of equid (horse and donkey) densities. Assessing the vector competence for ASHV of species such as *C. oxystoma*, *C. enderleini* and *C. miombo* is essential to evaluate their roles in disease transmission. Mapping not only equid densities but also equid movement networks in Senegal would be of great help for decision makers, since combining vectors abundance maps with maps of equid densities and movements of equids would enable evaluation of AHS transmission risk. In a second step, combining transmission risk with AHS virus introduction risk would enable making recommendations in terms of early warning systems and vaccination policies.

## Conclusions

To our knowledge, this study is the first to map the distribution of five major species of *Culicoides* of veterinary interest in Senegal. The highest abundances of *Culicoides* were observed in the south and in the groundnut basin, whereas abundances were the lowest in northern Senegal. Abundance maps were produced using three different modelling approaches. RF models provided better estimates of abundances than GLM models and were not limited by interpolation distances contrary to kriging. Environmental and climatic variables of importance that influence the spatial distribution of species abundance were identified. It is now crucial to evaluate the vector competence of major species and then combine the vector densities with densities of horses and ruminants to quantify the risk of transmission of AHS and BT virus across the country.

## Additional files

Additional file 1: **Table S1. ***Culicoides* data from Senegal throughout a nation-wide trapping campaign in 2012. (XLSX 15 kb)

Additional file 2: **Figure S1.** Climatic and environmental data on Senegalese territory with a spatial resolution on 1 km^2^. *Abbreviations*: Av, Average; Min, Minimum; Max, Maximum; Dlst, Day land surface temperature; Nlst, Night land surface temperature; lc, landcover; NDVI, Normalized difference vegetation index; Host, livestock density. (PDF 308 kb)

## Abbreviations

AHSV: African horse sickness virus
BTV: Bluetongue virus
DLST: Day-time land surface temperature
GLM: Generalized Linear Models
NDVI: Normalized difference vegetation index
NLST: Night-time land surface temperature
RF: Random Forest
