# Supplementary figures and images for: Spatial distribution modelling of Culicoides (Diptera: Ceratopogonidae) biting midges, potential vectors of African horse sickness and bluetongue viruses in Senegal
Source: Parasit Vectors. 2018 Jun 8;11:341. doi: 10.1186/s13071-018-2920-7 (PMC5994048; doi:10.1186/s13071-018-2920-7)

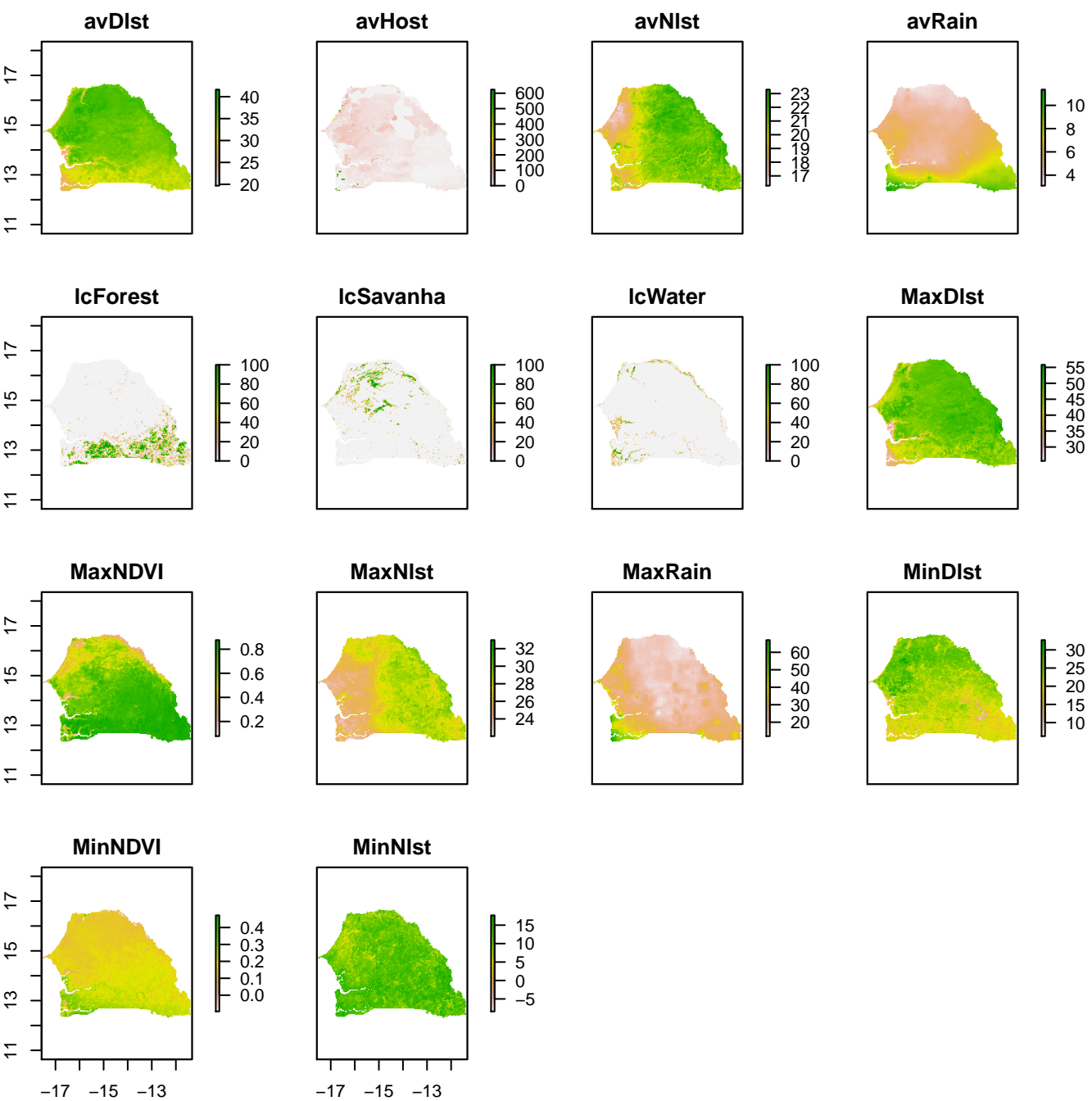

Supplement: Supplementary file 2 — Figure S1. Climatic and environmental data on Senegalese territory with a spatial resolution on 1 km2. Abbreviations: Av, Average; Min, Minimum; Max, Maximum; Dlst, Day land surface temperature; Nlst, Night land surface temperature; lc, landcover; NDVI, Normalized difference vegetation index; Host, livestock density. (PDF 308 kb) [file 13071_2018_2920_MOESM2_ESM.pdf]
